# LIMK2 Mediates Resistance to Chemotherapeutic Drugs in Neuroblastoma Cells through Regulation of Drug-Induced Cell Cycle Arrest

**DOI:** 10.1371/journal.pone.0072850

**Published:** 2013-08-21

**Authors:** Cristina Gamell, Alice V. Schofield, Randy Suryadinata, Boris Sarcevic, Ora Bernard

**Affiliations:** 1 Cytoskeleton and Cancer Unit, St. Vincent’s Institute of Medical Research, Melbourne, Victoria, Australia; 2 Department of Medicine at St. Vincent’s Hospital, The University of Melbourne, Melbourne, Victoria, Australia; 3 Cell Cycle and Cancer Unit, St. Vincent’s Institute of Medical Research, Melbourne, Victoria, Australia; Vanderbilt University Medical Center, United States of America

## Abstract

Drug resistance is a major obstacle for the successful treatment of many malignancies, including neuroblastoma, the most common extracranial solid tumor in childhood. Therefore, current attempts to improve the survival of neuroblastoma patients, as well as those with other cancers, largely depend on strategies to counter cancer cell drug resistance; hence, it is critical to understand the molecular mechanisms that mediate resistance to chemotherapeutics. The levels of LIM-kinase 2 (LIMK2) are increased in neuroblastoma cells selected for their resistance to microtubule-targeted drugs, suggesting that LIMK2 might be a possible target to overcome drug resistance. Here, we report that depletion of LIMK2 sensitizes SHEP neuroblastoma cells to several microtubule-targeted drugs, and that this increased sensitivity correlates with enhanced cell cycle arrest and apoptosis. Furthermore, we show that LIMK2 modulates microtubule acetylation and the levels of tubulin Polymerization Promoting Protein 1 (TPPP1), suggesting that LIMK2 may participate in the mitotic block induced by microtubule-targeted drugs through regulation of the microtubule network. Moreover, LIMK2-depleted cells also show an increased sensitivity to certain DNA-damage agents, suggesting that LIMK2 might act as a general pro-survival factor. Our results highlight the exciting possibility of combining specific LIMK2 inhibitors with anticancer drugs in the treatment of multi-drug resistant cancers.

## Introduction

Neuroblastoma is the most common extracranial solid tumor in childhood and the most frequently diagnosed malignancy during infancy [1]. Despite significant advances in our understanding of the etiology of this cancer, the outcome for children with a high-risk clinical manifestation has improved only modestly, with long-term survival being less than 40% [2,3]. This places neuroblastoma as one of the greatest challenges in pediatric oncology. Most neuroblastomas initially respond to chemotherapy and local radiotherapy, however neuroblastoma frequently relapses and becomes drug resistant [4]. Thus, it is of utmost importance to better understand the mechanisms that mediate resistance to chemotherapeutic drugs in order to develop strategies to combat drug-resistant cancers.

Anti-mitotic drugs that target microtubules, such as the vinca alkaloids, are extensively used for treating neuroblastoma and other pediatric malignances [5]. Microtubule-targeted drugs bind to and affect microtubule stability and dynamics [6], causing activation of the spindle assembly checkpoint and a delay or block at the metaphase-anaphase transition that can lead to cell death [7].

Resistance of neuroblastoma cells to microtubule-targeted drugs is attributed to overexpression of multi-drug resistance proteins such as the transmembrane efflux pump P-glycoprotein and the MDR-associated proteins [8–12] as well as alterations in microtubule stability [13]. In neuroblastoma cells selected for their resistance to vincristine and colchicine, expression of LIM kinase 2 (LIMK2) is significantly increased [14]. Furthermore, LIMK2 may be a predictive marker of drug resistance as its elevated expression correlates with the resistance of human cancer cell lines to a wide range of chemotherapeutic drugs with different mechanisms of action [15]. However, the signaling pathways that associate high levels of LIMK2 and chemotherapeutic drug resistance are not fully understood.

LIMK2 belongs to the LIM kinase family of serine/threonine kinases, which includes LIMK1 and LIMK2. The LIMKs are key regulators of actin dynamics through phosphorylation and inactivation of the actin depolymerizing factor cofilin [16–19]. Both LIMK proteins are ubiquitously expressed in mouse tissues [20–22], however, their subcellular localization differs. LIMK1 is localized to focal adhesions, whereas LIMK2 is found in cytoplasmic puncta and at the perinuclear region in association with the cis-golgi compartment [20]. Their different subcellular localization suggests that their regulation and/or substrates might be different.

Two major LIMK2 transcripts are generated by alternative splicing, LIMK2a and LIMK2b [23]. LIMK2a represents the full-length transcript whereas LIMK2b encodes a protein lacking half of the first LIM domain, which is replaced by a random sequence. This replacement is unique to the LIMK2 gene and is conserved in mice and humans. Recent studies demonstrated that LIMK2b, but not LIMK2a, is a p53 target gene that is upregulated by DNA damage [24,25], however little else is known about the functional differences between these two proteins.

In this study, we report that LIMK2 acts as a survival factor in neuroblastoma cell lines to counteract the effect of diverse chemotherapeutic drugs and shed light on the signaling pathways that may associate LIMK2 with tumor cell resistance.

## Results

### High levels of LIMK2 lead to an increased number of multinucleated cells

**Figure 1:**
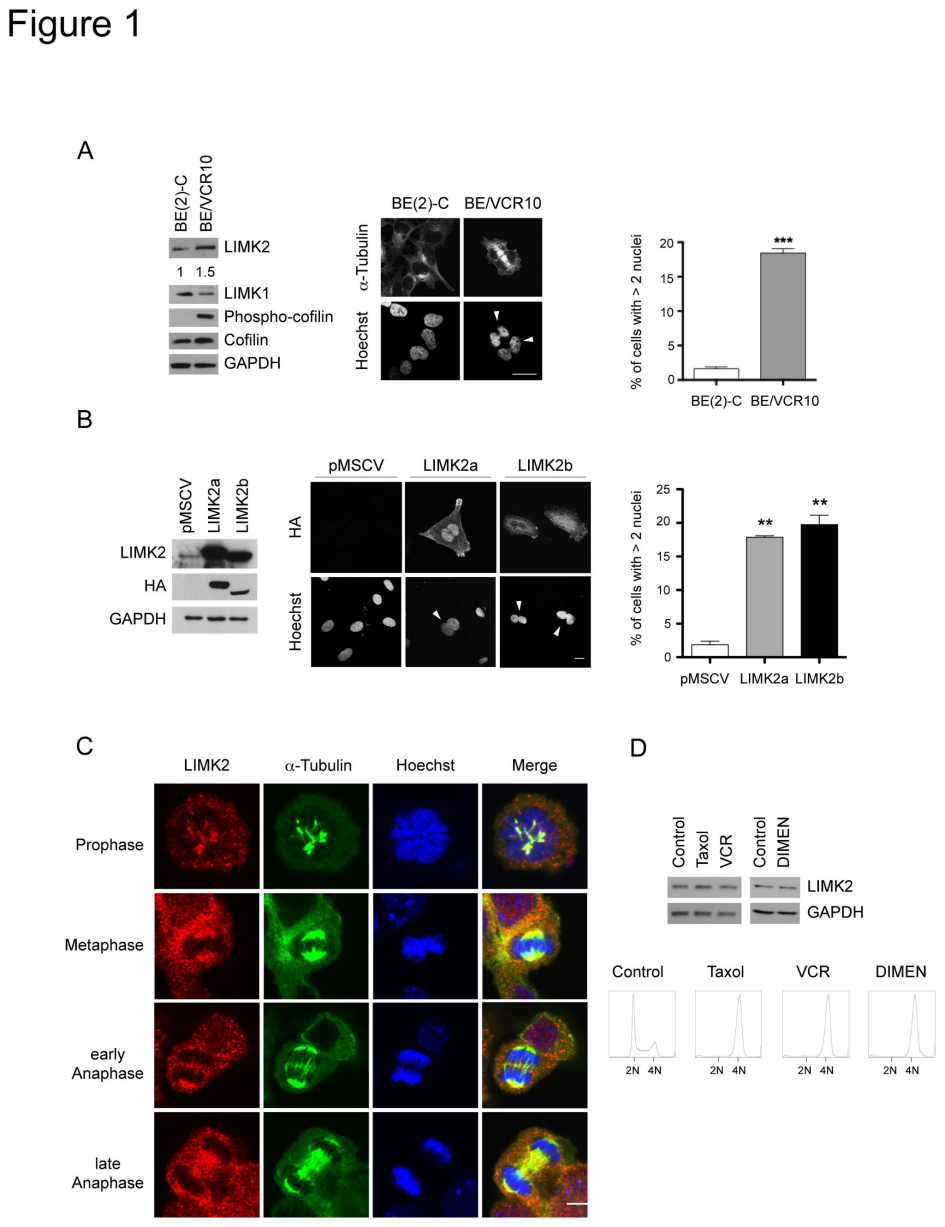
LIMK2 overexpression results in increased number of multinucleated cells. (A) A high percentage of BE/VCR10 cells are multinucleated. Fixed BE(2)-C and BE/VCR10 cells were stained with FITC-conjugated anti-α-tubulin antibody and Hoechst. White arrowheads indicate multinucleated cells. Scale bar = 20 µm. The percentage of multinucleated cells shown on the right panel is represented as mean ± S.E.M of three independent experiments (***, p < 0.0001, unpaired t-test). The blots on the left show whole cell lysates of the BE(2)-C and BE/VCR10 cells analyzed by immunoblotting with the indicated antibodies. The numbers below the top panel represent the fold changes in the indicated protein levels. (B) Overexpression of LIMK2a or LIMK2b proteins in SHEP cells increases ploidy. Stable SHEP cell lines expressing HA-tagged LIMK2a or LIMK2b or vector control (pMSCV) were generated by transduction with retroviruses. The left panel shows the relative expression of the ectopic LIMK2 proteins. Cells expressing LIMK2a or LIMK2b were fixed and stained with an anti-HA antibody and Hoechst (middle panel). White arrowheads indicate multinucleated cells. Scale bar = 20 µm. The percentage of multinucleated cells is shown on the right panel represented as mean ± S.E.M of three independent experiments (**, p < 0.001, unpaired t-test). (C) LIMK2 localizes to the mitotic spindle microtubules. An unsynchronized population of NIH-3T3 cells was fixed and immuno-stained with an anti-LIMK2 antibody (red), FITC-conjugated anti-α-tubulin antibody (green) and Hoechst (blue). Scale bar = 20 µm. (D) LIMK2 levels do not change during cell division. SHEP cells were treated with 0.5 µM taxol, 0.1 µM vincristine (VCR) or 1 µM dimethylenastron (DIMEN) for 20 hours and mitotic cells were collected by mitotic shake-off. Half of the cells were used to prepare whole cell lysates that were immunoblotted with anti-LIMK2 as well as anti-GAPDH antibodies. The rest of the cells were fixed, stained with propidium iodide and analyzed by flow cytometry (bottom panels). The cell cycle profiles show the G2/M arrest with the different treatments.

To understand the role of LIMK2 in drug resistance, we examined the effect of high LIMK2 levels on the morphology of BE(2)-C neuroblastoma cells selected for their resistance to vincristine (BE/VCR10). These cells showed a similar organization of filamentous actin and microtubules (data not shown), however approximately 20% of the BE/VCR10 cells were found to be multinucleated (Figure 1A). We therefore explored the possibility that this increased ploidy was due to high LIMK2 levels. We found that stable expression of LIMK2a and LIMK2b in SHEP neuroblastoma cells resulted in a significant increase in the percentage of multinucleated cells compared with vector expressing cells (Figure 1B), suggesting that the high LIMK2 expression in the BE/VCR10 cell line is responsible for their multinucleated phenotype.

The increased number of multinucleated cells in the LIMK2 overexpressing cells suggests that LIMK2 participates in cell division and/or cytokinesis. Immunostaining of mitotic cells for LIMK2 showed that LIMK2 co-localizes during metaphase and early-anaphase with the spindle microtubules and in late-anaphase it was found at the spindle midzone (Figure 1C), as previously described [26,27]. No difference in LIMK2 levels was observed in SHEP cells synchronized in mitosis compared with an asynchronous cell population (Figure 1D), suggesting that LIMK2 levels are not modulated during cell division. However, the observation that LIMK2 colocalizes with spindle microtubules, which are highly dynamic and are therefore very sensitive to the effects of microtubule-targeted drugs, supports a role for LIMK2 in microtubule-targeted drug-responsiveness.

### LIMK2 knockdown sensitizes cells to microtubule-targeted drugs

**Figure 2:**
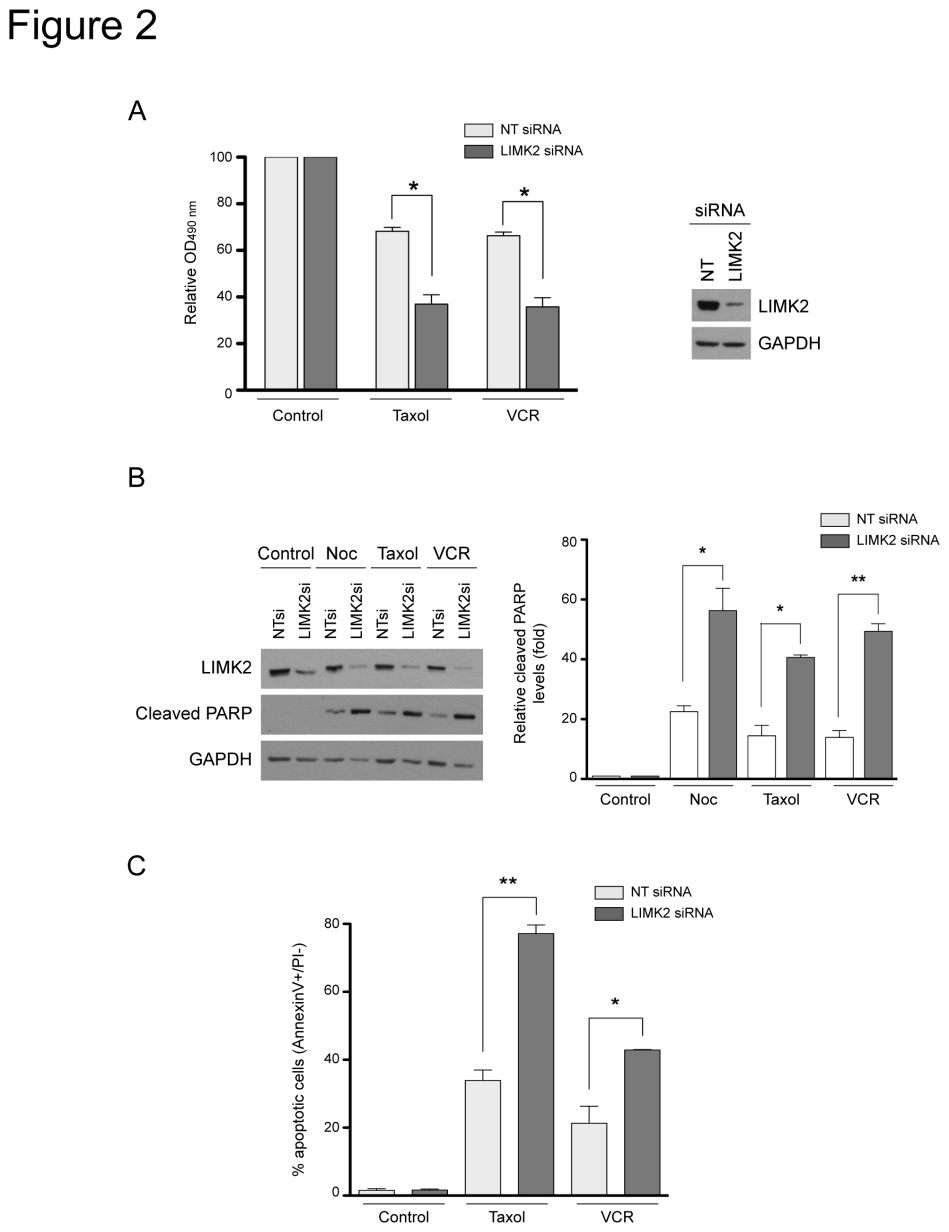
LIMK2 knockdown sensitizes cells to apoptosis induced by microtubule-targeted drugs. (A) LIMK2 knockdown increases the sensitivity of SHEP cells to microtubule-targeted drugs. SHEP cells were transfected with LIMK2 or non-targeting control (NT) siRNA. 72 hours after transfection, cells were treated with 0.5 µM taxol or 0.1 µM vincristine (VCR) for 48 hours and cell viability was analyzed by MTT assay. Results are expressed as percentage of the control (vehicle) and represented as mean ± S.E.M of three independent experiments (*, p < 0.05, unpaired t-test). The efficiency of the LIMK2 knockdown in a representative experiment is shown on the right panel. (B) LIMK2-depleted cells show enhanced apoptosis induced by microtubule-targeted drugs. SHEP cells transfected with the indicated siRNAs were treated with 0.5 µg/ml nocodazole (Noc), 0.5 µM taxol or 0.1 µM vincristine (VCR) for 24 hours and analyzed by immunoblots probed with the indicated antibodies. The graph on the right that indicates the relative cleaved PARP levels compared to control (vehicle) as mean ± S.E.M of three independent experiments (*, p < 0.05; **, p < 0.001, unpaired t-test). (C) SHEP cells were transfected with the indicated siRNAs and treated with 0.5 µM taxol or 0.1 µM vincristine (VCR) for 72 hours. Apoptosis was determined by flow cytometry and is shown as the mean percentage of AnnexinV+/PI- cells ± S.E.M of three independent experiments (*, p < 0.05; **, p < 0.001, unpaired t-test).

To establish the functional relevance of LIMK2 in the cellular response to microtubule-targeted drugs, we analyzed the consequence of LIMK2 knockdown on the effect of several drugs on cell viability. We have used the SHEP neuroblastoma cells in these experiments since, unlike the BE(2)-C neuroblastoma cells, they express barely detectable levels of the multidrug transporter, P-glycoprotein [28]. In the absence of drugs, transfection with LIMK2 specific- or with non-targeting-siRNA did no affect cell viability compared to untransfected cells (data not shown). However, LIMK2-depleted cells showed increased sensitivity to the microtubule-targeted drugs taxol and vincristine compared to control siRNA-transfected cells (Figure 2A). This increased sensitivity correlated with enhanced apoptosis in response to microtubule-targeted drugs, as demonstrated by the increased levels of cleaved PARP (Figure 2B). Consistently, flow cytometry analysis of cells stained with AnnexinV and propidium iodide also showed that down-regulation of LIMK2 sensitized cells to drug-induced apoptosis (Figure 2C).

### LIMK2 regulates the cell cycle arrest induced by microtubule-targeted drugs

**Figure 3:**
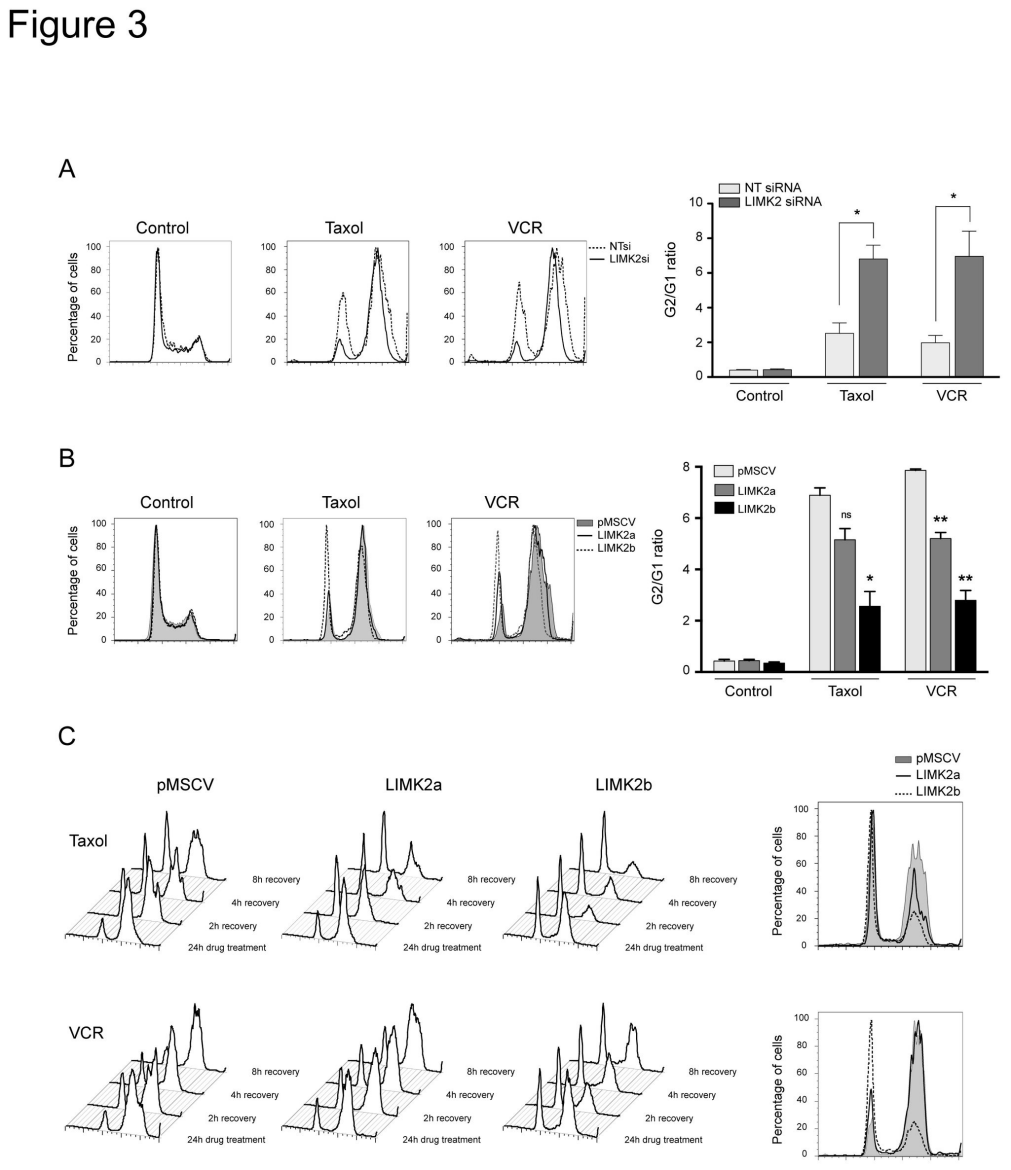
LIMK2 regulates microtubule drug-dependent cell cycle arrest. (A) LIMK2 knockdown enhances the G2/M block induced by microtubule-targeted drugs. Cell cycle analysis of SHEP cells transfected with LIMK2 or non-targeting control (NT) siRNA for 72 hours followed by treatment with 0.5 µM taxol or 0.1 µM vincristine (VCR) for 24 hours. The extent of the G2/M arrest, represented by G2/G1 ratio, is shown on the right as mean ± S.E.M of three independent experiments (*, p < 0.05, unpaired t-test). (B) LIMK2 overexpressing cells reduce the G2/M arrest induced by microtubule-targeted drugs. Cell cycle analysis of LIMK2a or LIMK2b expressing cells treated with 0.5 µM taxol or 0.1 µM vincristine (VCR) for 24 hours. The extent of the G2/M arrest, represented by G2/G1 ratio, is shown on the right as mean ± S.E.M of three independent experiments (*, p < 0.05; **, p < 0.001; ns, non significant, unpaired t-test). (C) LIMK2 overexpressing cells recover faster from the cell cycle block induced by microtubule-targeted drugs. Analysis of the cell cycle recovery after drug wash-out of SHEP cells expressing LIMK2a or LIMK2b treated with 0.5 µM taxol or 0.1 µM vincristine (VCR) for 24 hours. The right panels show the cell cycle profile of the cells 8 hours after drug removal.

We then explored whether LIMK2 was involved in the mitotic block induced by the microtubule-targeted drugs. Silencing LIMK2 in untreated cells did not affect the cell cycle profile compared to siRNA-transfected controls (Figure 3A), supporting our hypothesis that LIMK2 is not involved in the normal cell cycle. However, in the presence of microtubule-targeted drugs, LIMK2-depleted cells exhibited a dramatic increase in the G2/M population compared with the controls (Figure 3A).

To further study the involvement of LIMK2 in the microtubule-targeted drug-induced mitotic arrest, we analyzed the effect of taxol and vincristine on the LIMK2a and LIMK2b overexpressing SHEP cells. As expected, while treatment of control cells with microtubule-targeted drugs resulted in increased percentage of G2/M arrested cells (Figure 3B), overexpression of LIMK2b, and to a lesser extent LIMK2a, greatly reduced the number of G2/M arrested cells (Figure 3B). We also analyzed the recovery of LIMK2-overexpressing cells after drug-induced mitotic block by washing-out the drugs and allowing them to re-enter the cell cycle. Eight hours after drug removal, most of the control cells were still arrested in G2/M. In contrast, overexpression of LIMK2a or LIMK2b accelerated the recovery rate of cells arrested at mitosis, with a more efficient recovery of cells overexpressing LIMK2b (Figure 3C). These results demonstrate that increased LIMK2 levels have an impact on the cell cycle arrest induced in response to disruption of microtubule dynamics.

### LIMK2 modulates the levels of tubulin polymerization promoting protein 1 (TPPP1) levels and microtubule acetylation

**Figure 4:**
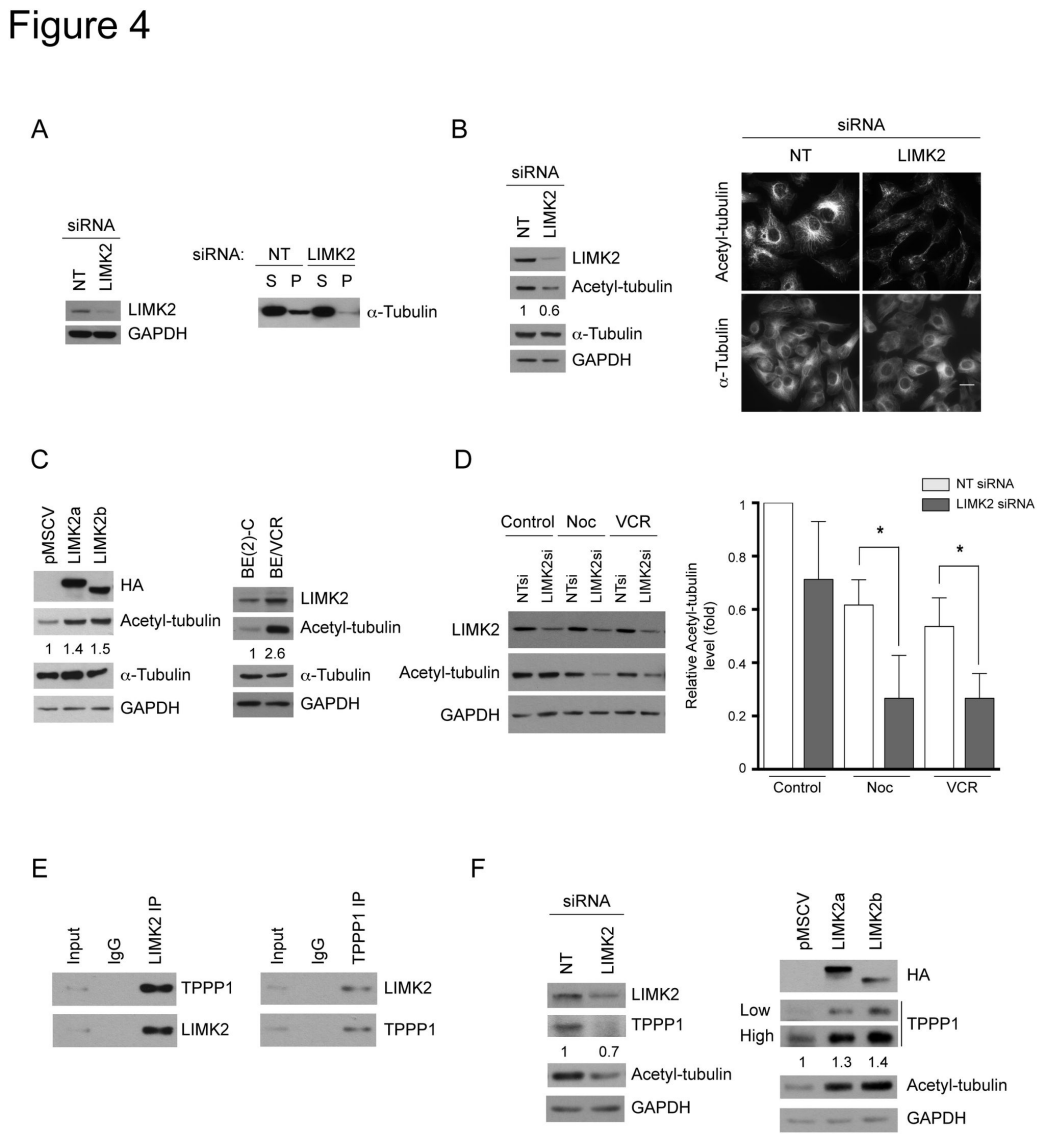
LIMK2 affects microtubule acetylation and TPPP1 levels. (A) LIMK2-depleted cells have reduced amounts of polymerized tubulin. SHEP cells were transfected with LIMK2 or non-targeting control (NT) siRNA and 72 hours later the soluble (S) and polymerized (P) tubulin fractions were separated by centrifugation and analyzed by immunoblotting. A representative immunoblot of three experiments is shown. The efficiency of the LIMK2 knockdown and the loading control is shown on the left panel. (B) LIMK2 knockdown cells show reduced levels of acetylated tubulin. The numbers below the second panel represent the relative level of acetylated tubulin. Cells transfected with the indicated siRNAs were analyzed by immunoblotting and immunofluorescent staining. Bar = 20 µm. (C) SHEP cells overexpressing LIMK2 have increased levels of acetylated tubulin. SHEP cells overexpressing LIMK2a and LIMK2b as well as BE(2)-C and BE/VCR10 cells were analyzed by immunoblotting. The numbers below the second panel represent the relative level of acetylated tubulin. (D) LIMK2 knockdown cells are more sensitive to microtubule depolymerization induced by microtubule-targeted drugs. SHEP cells transfected with the indicated siRNAs were treated with 0.5 µg/ml nocodazole (Noc) or 0.1 µM vincristine (VCR) for 24 hours and analyzed by immunoblotting. The graph indicates the relative acetylated tubulin levels compared with the NT siRNA control (vehicle) represented as mean ± S.E.M of three independent experiments (*, p < 0.05). (E) LIMK2 and TPPP1 interact in SHEP cells. LIMK2 or TPPP1 were immunoprecipitated from SHEP cell lysates and the respective co-immunoprecipitated TPPP1 (left panel) or LIMK2 (right panel) were detected by immunoblotting. (F) LIMK2 modulates TPPP1 protein levels. Lysates from SHEP cells transfected with LIMK2 or control (NT) siRNA (left panels) and LIMK2a or LIMK2b overexpressing SHEP cells (right panels) were analyzed by immunoblotting. Two different exposures of TPPP1 immunoblot are shown (low and high). The numbers below the top panels in B, C, and F represent the folds change in the indicated protein levels.

Resistance to microtubule-targeted drugs is associated with microtubule stability [29]. We therefore examined whether the increased sensitivity to microtubule-targeted drugs of the LIMK2-depleted cells correlated with reduced stability of their microtubule network. Indeed, the amount of polymerized tubulin in LIMK2-depleted cells was lower compared with the control (Figure 4A).

Acetylated microtubules are resistant to drug-induced microtubule depolymerization [30]. They also have a very long half life; therefore, an increase in microtubule acetylation is associated with stabilization of the microtubule network [31]. Consistent with the low levels of polymerized tubulin observed in the LIMK2-depleted cells, they also have reduced levels of acetylated tubulin as assessed by immunoblotting and immunofluorescence (Figure 4B). Conversely, SHEP cells overexpressing LIMK2a or LIMK2b contain increased levels of acetylated tubulin (Figure 4C). In agreement with a previous report showing that BE/VCR10 cells have increased amounts of polymerized tubulin compared with the parental BE(2)-C cells [13], we demonstrated here that their acetylated tubulin levels are increased as well (Figure 4C). Furthermore, the reduced levels of acetylated microtubules of the LIMK2-depleted cells correlated with an enhanced sensitivity to microtubule-targeted drug-induced depolymerization. Therefore, LIMK2 knockdown and treatment with the microtubule-destabilizing drugs nocodazole or vincristine synergistically reduced acetylated tubulin levels (Figure 4D). Overall, these results suggest that LIMK2 affects sensitivity to microtubule-targeted drugs through modulation of microtubule acetylation.

The question remains as to how LIMK2 regulates microtubule acetylation. It has been reported recently that LIMK2 modulates astral microtubules dynamics via tubulin polymerization promoting protein 1 (TPPP1) [26]. TPPP1 promotes microtubule polymerization [32,33] and increases tubulin acetylation via its interaction with and inhibition of histone deacetylase 6 (HDAC6), a major α-tubulin deacetylase [34]. We therefore hypothesized that LIMK2 regulates tubulin acetylation through modulation of TPPP1. We demonstrated that the endogenous LIMK2 and TPPP1 proteins interact in neuroblastoma cells (Figure 4E), as previously demonstrated in HeLa cells overexpressing LIMK2 and TPPP1 [26]. Furthermore, LIMK2 depletion, which results in reduced acetylated tubulin levels, was accompanied by decreased TPPP1 levels (Figure 4F); while overexpression of LIMK2a or LIMK2b correlated with increased TPPP1 levels (Figure 4F), suggesting that the effects of LIMK2 on tubulin acetylation could be mediated through TPPP1.

### LIMK2 participates in DNA damage response

**Figure 5:**
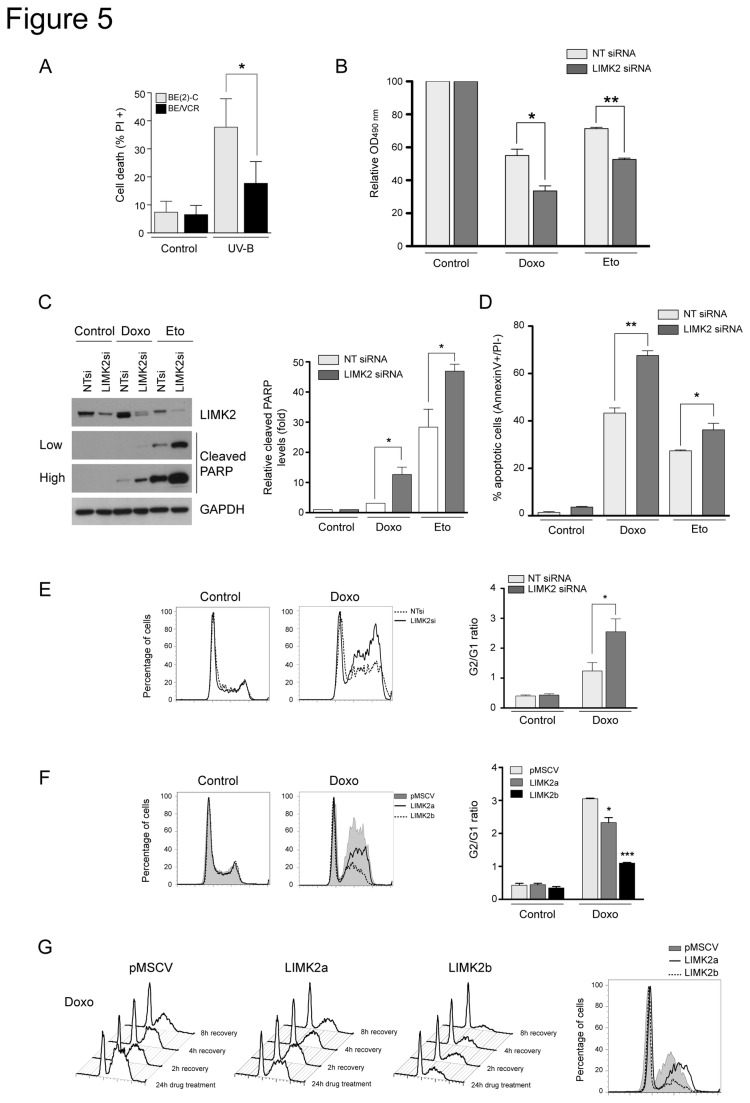
LIMK2 participates in DNA damage response. (A) BE/VCR10 cells are more resistant to genotoxic stress compared with the BE(2)-C parental cell line. Confluent monolayers of the BE(2)-C and BE/VCR10 cells were irradiated with 8 mJ/cm^2^ ultraviolet B (UV-B). After 24 hours recovery, adherent and non-adherent cells were collected, stained with propidium iodide and analyzed by flow cytometry. Cell death is represented as the mean percentage of propidium iodide positive cells ± S.E.M of three independent experiments (*, p < 0.05, unpaired t-test) (B) Knockdown of LIMK2 in SHEP cells increases their sensitivity to genotoxic stress. SHEP cells were transfected with LIMK2 or non-targeting control (NT) siRNA and 72 hours later they were treated with 10 µM doxorubicin (Doxo) or 10 µM etoposide (Eto) for 48 hours before cell viability was analyzed by MTT assay. Results are expressed as percentage of the control (vehicle). Mean ± S.E.M of three independent experiments (*, p < 0.05; **, p < 0.001, unpaired t-test). (C) LIMK2 knockdown sensitizes cells to apoptosis induced by DNA damage agents. SHEP cells transfected with the indicated siRNAs were treated with 10 µM doxorubicin (Doxo) or 100 µM etoposide (Eto) for 24 hours and analyzed by immunoblotting. Two different exposures of the cleaved PARP immunoblot are shown (low and high). The graph indicates the relative cleaved PARP levels compared to control (vehicle) and is represented as the mean ± S.E.M of three independent experiments (*, p < 0.05, unpaired t-test). (D) SHEP cells transfected with the indicated siRNAs were treated as in A for 72 hours and apoptosis was analyzed by flow cytometry. The percentage of apoptotic cells is represented by the mean percentage of AnnexinV+/PI- cells ± S.E.M of three independent experiments (*, p < 0.05; **, p < 0.001, unpaired t-test). (E) LIMK2 knockdown promotes G2/M arrest induced by DNA damage. Cell cycle analysis of SHEP cells transfected with the indicated siRNAs and treated with 10 µM doxorubicin (Doxo) for 24 hours. The G2/G1 ratio of three independent experiments is shown on the right as the mean ± S.E.M (*, p < 0.05, unpaired t-test). (F) Cell cycle analysis of LIMK2a or LIMK2b overexpressing cells treated with 10 µM doxorubicin (Doxo) for 24 hours. The extent of the G2/M arrest, represented by G2/G1 ratio, is shown on the right as mean ± S.E.M of three independent experiments (*, p < 0.05; ***, p < 0.0001, unpaired t-test). (G) LIMK2 overexpressing cells are more resistant to DNA damage-induced cell cycle arrest. SHEP cells expressing LIMK2a or LIMK2b were treated with 10 µM doxorubicin (Doxo) for 24 hours before washing out the drugs and the cell cycle recovery was analyzed by flow cytometry. The panel on the right depicts the cells cycle profile 8 hours after drug removal.

Because high levels of LIMK2 correlate with resistance to microtubule-targeted drugs and to chemotherapeutic drugs with other modes of action [15], we hypothesized that LIMK2 might be a common contributor to chemo-resistance. We first examined whether the vincristine-resistant BE/VCR10 cells expressing high levels of LIMK2 were also more resistant to DNA damage. As mentioned above, since the BE(2)-C cells and the drug-resistant subline express high levels of the multidrug transporter P-glycoprotein [28], genotoxic stress was induced by ultraviolet B irradiation. We found that the BE/VCR10 cells were also more resistant to ultraviolet B irradiation-induced cell death compared to the parental cell line (Figure 5A). We next analyzed the effect of LIMK2 knockdown on the sensitivity of SHEP cells (which express very low levels of the multidrug transporter P-glycoprotein in comparison with the BE/VCR10 cells [28]) to DNA damage agents. Silencing LIMK2 significantly increased the sensitivity of cells to doxorubicin and etoposide similar to the effect observed with the microtubule-targeted drugs (Figure 5B). Moreover, this enhanced sensitivity correlated with increased drug-induced apoptosis (Figure 5C and D). Furthermore, LIMK2 knockdown significantly increased the G2/M block induced by doxorubicin compared to the control cells, suggesting that this increase in drug sensitivity is due to an enhanced cell cycle arrest (Figure 5E). Conversely, overexpression of LIMK2a or LIMK2b conferred resistance to the doxorubicin-induced G2/M arrest as well as an accelerated cell cycle recovery after drug removal compared with control cells (Figure 5F and G). Taken together, these data demonstrate that LIMK2 is also involved in the DNA damage response and further supports our hypothesis that LIMK2 is a pro-survival factor.

### LIMK2 levels are upregulated by DNA damage agents but not by microtubule-targeted drugs

**Figure 6:**
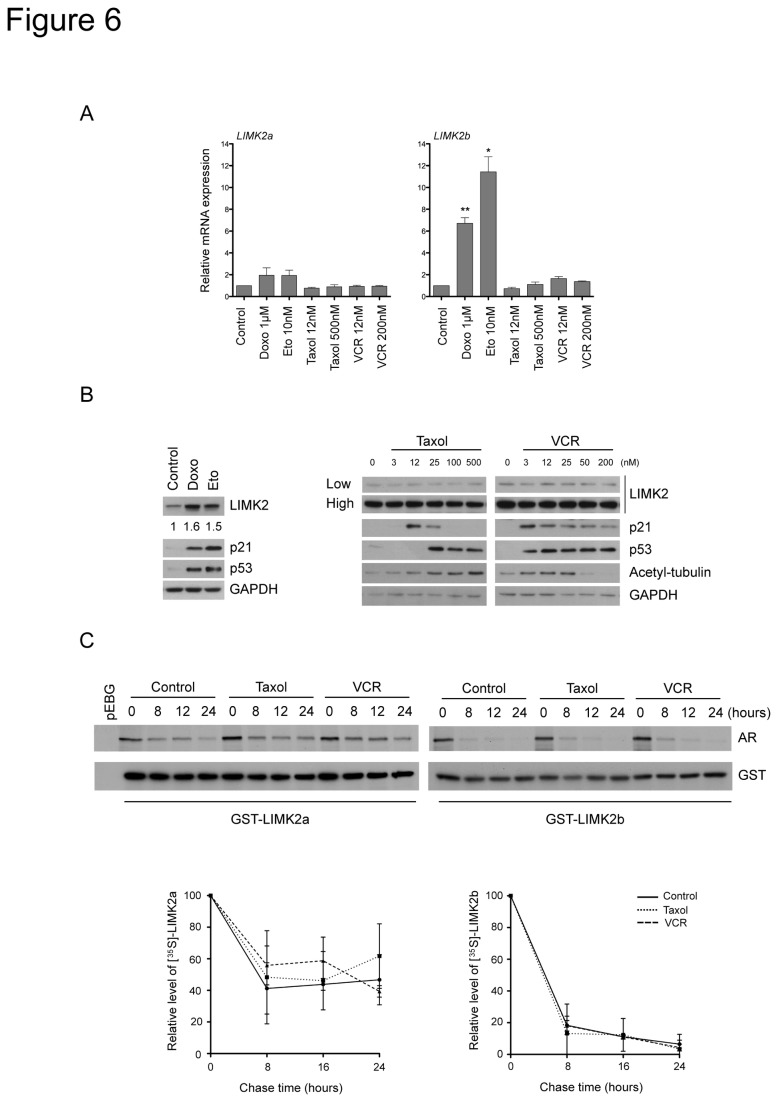
LIMK2 levels are upregulated by DNA damage agents but not by microtubule-targeted drugs. (A) LIMK2 expression is not regulated at the transcriptional level by microtubule-targeted drugs. SHEP cells were treated with the indicated drugs for 16 hours. The levels of LIMK2a and LIMK2b mRNA were quantified by qRT-PCR, normalized to the housekeeping gene L32, and plotted as relative expression to the control ± S.E.M of three independent experiments (*, p < 0.05; **, p < 0.001, unpaired t-test). (B) LIMK2 protein levels are induced by genotoxic stress but not by treatment with microtubule-targeted drugs. SHEP cells were treated with 1 µM doxorubicin (Doxo), 10 µM etoposide (Eto) or with taxol or vincristine (VCR) at the indicated concentrations for 24 hours before immunoblotting analysis. Two different exposures of LIMK2 immunoblot are shown (low and high). The numbers below the top panel represent the fold-change in the indicated protein levels. (C) Microtubule-targeted drugs do not affect the stability of LIMK2a or LIMK2b proteins. SHEP cells were transiently transfected with GST-LIMK2a or GST-LIMK2b and 24 hours later, they were incubated with 25 nM taxol or 2 nM vincristine (VCR) for 10 hours. Cells were then pulse-labeled with [^35^S]-methionine/cysteine and chased in the presence of the microtubule-targeted drugs for the indicated time periods. The GST-tagged proteins were purified with glutathione sepharose beads and analyzed by autoradiography (AR) and immunoblotting with anti-GST antibody. Quantification of the level of GST-LIMK2a and GST-LIMK2b proteins from three separate assays is expressed as mean ± S.E.M of percentage values of the samples at zero time.

Since LIMK2b, but not LIMK2a, is a p53-transcriptional target gene induced by genotoxic stress that promotes cell survival [24,25], we examined if microtubule-targeted drugs also induced LIMK2 transcription. Low doses of microtubule-targeted drugs, which suppress microtubule dynamics without affecting the microtubule polymer mass, enhance p53 accumulation in the nucleus and activation of p53 target genes [35–39]. In contrast, disruption of the microtubule network by treatment with high concentration of microtubule-targeted drugs impedes p53 translocation to the nucleus and in turn inhibits activation of p53 targets [35–39]. As previously shown in other cancer cell lines [24,25], treatment of SHEP cells (p53 wild-type) with DNA damage agents increased LIMK2b mRNA levels but not that of LIMK2a (Figure 6A). However, treatment with low or high concentrations of microtubule-targeted drugs had no effect on LIMK2a or LIMK2b transcript levels (Figure 6A).

To investigate whether microtubule-targeted drugs increased LIMK2 protein levels by a post-transcriptional mechanism, we analyzed the effect of these drugs on LIMK2 protein levels. While increased LIMK2b mRNA levels in cells treated with DNA damage agents correlated with elevated LIMK2 protein and increased levels of the p53 target gene p21, treatment with microtubule-targeted drugs did not alter LIMK2 protein levels (Figure 6B) or protein stability (Figure 6C), therefore showing that chemotherapeutic drugs with different modes of action have a different impact on LIMK2 levels. Interestingly, we found that the LIMK2a protein was extremely stable with a half-life of ^~^24 h, while the half-life LIMK2b was only ^~^6 h (Figure 6C). The implication of this difference in LIMK2 proteins stability remains to be determined but it may potentially explain the distinct effects of LIMK2a and LIMK2b overexpression on the drug-induced cell cycle arrest (Figures 3 and 5).

## Discussion

The data presented here shed light on the role of LIMK2 in the sensitivity of cells to chemotherapeutic drugs. We establish that LIMK2 acts as a pro-survival factor in response to several microtubule-targeted drugs and DNA damage agents, supporting the hypothesis that LIMK2 is a common contributor to drug resistance. Importantly, down-regulation of LIMK2 levels sensitizes cells to chemotherapeutic drugs, which is likely to be the consequence of an enhanced mitotic arrest leading to increased apoptosis. Moreover, our findings suggest that LIMK2 participates in the drug-induced cell cycle block by regulating the microtubule network.

We propose that LIMK2 is required for a proper cell cycle arrest after treatment with chemotherapeutic drugs. This conclusion is based on the following observations: (i) alteration of LIMK2 levels has a profound impact on the mitotic block induced by chemotherapeutic drugs, (ii) LIMK2 colocalizes with spindle microtubules, (iii) overexpression of LIMK2 increases the percentage of multinucleated cells and (iv) altered LIMK2 levels impact the stability of the microtubule network. These findings are consistent with a previous study showing that LIMK2 down-regulation induces abnormal mitotic spindles and that this effect is enhanced in the presence of microtubule-targeted drugs [14].

Here, we provide evidence that LIMK2 knockdown enhances cell sensitivity to several microtubule-targeted drugs with different mechanisms of action, suggesting that LIMK2 plays a central role in cellular processes that promote cell survival. The role of LIMK2 as a general pro-survival factor is further highlighted by the observation that certain DNA damage agents induce an increase in LIMK2 levels in SHEP cells, which promotes cell survival. The role of LIMK2 in the regulation of cell sensitivity to microtubule-targeted drugs was previously reported by Po’uha et al. [14]; however, while we have found that LIMK2 participates in resistance to microtubule-stabilizing and -destabilizing drugs, Po’uha et al. showed that knockdown of LIMK2 does not significantly alter the sensitivity of SHEP cells to the microtubule-stabilizing drugs taxol or epothilone. A likely explanation for the discrepancies between the two studies may be due to the difference in the methodologies. While we used MTT assays to analyze the effect of LIMK2 on drug sensitivity, Po’uha et al. performed clonogenic assays.

The data presented here clearly show that the apoptotic cell death induced by microtubule-targeted drugs and DNA damage agents is increased in LIMK2-depleted cells. The increased cell sensitivity to chemotherapeutic drugs is due to enhanced apoptosis potentially as a consequence of an increased/prolonged cell cycle arrest in the G2/M phase. These findings are in line with previous studies showing a role for LIMK2 in radiation-induced cell cycle arrest [24,25]. Hsu et al. reported that depletion of LIMK2 promoted early exit from the G2/M arrest after DNA damage [25], however Croft et al. concluded that it prolonged the arrest [24]. In agreement with Croft et al., we show that in response to doxorubicin, silencing LIMK2 enhances the G2/M block, whereas LIMK2 overexpression confers resistance to doxorubicin-induced cell cycle arrest.

Interestingly, LIMK2b overexpressing cells showed a profound resistance to drug-induced cell cycle arrest, whereas the effect of LIMK2a overexpression was less pronounced. Consistent with previous findings showing that LIMK2b is a p53-target gene upregulated in response to genotoxic stress [24,25], we demonstrate here that treatment of neuroblastoma cells with the DNA damage agents doxorubicin or etoposide induces an increase in the LIMK2b transcript levels without affecting LIMK2a. The difference between LIMK2a and LIMK2b proteins may be afforded by the unique LIMK2b N-terminus that may have a different binding motif to that of LIMK2a, thus promoting or disrupting its interaction with different proteins and thereby participating in divergent signaling pathways. Notably, we show here for the first time that the stability of the LIMK2a protein is different from that of LIMK2b. LIMK2a is an extremely stable protein with a half-life of ^~^24 hours, similar to that of LIMK1 [40]. In contrast, LIMK2b has a much shorter half-life of only ^~^6 hours. Since binding of the chaperone protein Hsp90 to LIMK1 or LIMK2 promotes the formation of homodimers and their subsequent trans-phosphorylation, resulting in their increased stability [40], it is possible that the changes in the N-terminal of the LIMK2b protein interferes with its ability to interact with Hsp90 and form homodimers, which could reduce its stability.

While the effect of LIMK2 on the chemotherapeutic drug-induced cell cycle arrest is apparent, the signaling pathways that govern this link are less clear. We propose that LIMK2 participates in the mitotic block by regulating the stability of the microtubule network, and that LIMK2 exerts these effects via modulation of TPPP1 levels. This is supported by our recently published findings showing that TPPP1 regulates cell cycle progression through modulation of microtubule acetylation and dynamics [41]. It was previously suggested that the interaction between LIMKs and TPPP1 results in TPPP1 phosphorylation [26,42], however studies from our group clearly demonstrated that TPPP1 is a Rho kinase (ROCK) substrate and that it is not phosphorylated by LIMK1 or LIMK2 [43].

In summary, this study shows that high LIMK2 levels correlate with resistance to a wide range of chemotherapeutic drugs through different mechanisms of action and that LIMK2 down-regulation increases the sensitivity of neuroblastoma cells to these drugs. Moreover, we demonstrate that this increase in drug sensitivity correlates with enhanced cell cycle arrest and apoptosis. Further studies are required to examine the relationship between LIMK2 expression and drug resistance in a broad panel of cell lines representative of high-risk neuroblastoma as well as in tumor samples from neuroblastoma patients taken before and after chemotherapy. The most exciting conclusion of our study is that LIMK2 is a common contributor to chemotherapeutic drug resistance, suggesting that combining specific LIMK2 inhibitors with certain chemotherapeutic agents may be an attractive strategy for the treatment of drug-resistant neuroblastomas.

## Materials and Methods

### Cell cultures

The BE(2)-C [28], BE/VCR10 [28] and SHEP [14] cell lines were a kind gift of Dr Maria Kavallaris (Lowy Cancer Research Centre, Sydney, Australia). NIH-3T3 and HEK293T were acquired from ATCC (www.atcc.org). All cells were maintained in Dulbecco’s Modified Eagles Medium (DMEM) (Sigma) supplemented with 10% fetal bovine serum (FBS), 100 U/ml penicillin and 100 µg/ml streptomycin at 37°C in a humidified 5% CO_2_ incubator.

SHEP cell lines expressing LIMK2a, LIMK2b or vector were generated by infection with amphotropic retroviruses. HEK293T cells were co-transfected with expression constructs and the amphotropic helper plasmid at a 1:4 ratio using Fugene 6 (Roche). 8 hours later the medium was replaced with fresh DMEM without phenol red (GIBCO) supplemented with 10% FBS and 24, 48 and 72 hours post-transfection virus containing supernatants were collected, filtered and concentrated. SHEP cells were infected with viral supernatant supplemented with 4 µg/ml Polybrene (Sigma) by spin-inoculation at 1300 x g for 1 hour. Transduced cells expressing the MSCV-Cherry constructs were isolated using a FACSAria flow cytometer (BD Biosciences) at day 14 after infection.

### Reagents and Treatments

Taxol was purchased from Cytoskeleton and nocodazole, doxorubicin and etoposide from Sigma. Dimethylenastron and vincristine were a gift from Dr Maria Kavallaris (Lowy Cancer Research Centre, Sydney, Australia).

For ultraviolet B irradiation, cells were irradiated in uncovered tissue culture plates with 8 mJ/cm^2^ using a UV crosslinker (Uvlink CL-508). After 24 hours recovery, cell death was analyzed by flow cytometry.

### RNA interference assays

SHEP cells were transfected with ON–TARGETplus SMARTPool (Dharmacon) hLIMK2 or non-targeting siRNA using Lipofectamine 2000 (Invitrogen). Knockdown was analyzed by immunoblotting 72 hours post-transfection.

### Immunoblotting and immunoprecipitation

Immunoblot analysis was performed as described previously [44]. Antibodies to cleaved PARP (#9541), phospho-cofilin (Ser3, #3311) and GAPDH-HRP (#3683) were from Cell Signaling. Anti-α-tubulin (T5168) and anti-acetyl-tubulin (T6793) were from Sigma. Additional antibodies used were anti-HA (Roche, 11867423001), anti-LIMK1 [(clone 8D5–5-12 [21]], anti-LIMK2 (Abcam, ab45165), TPPP1 [42], anti-p21 (Santa Cruz, sc-397), anti-cofilin (Cytoskeleton, ACFL02) and p53 (gift from Dr Ygal Haupt, Peter MacCallum Cancer Centre, Melbourne, Australia).

The effect of LIMK2 siRNA on the polymerized mass of microtubules was determined as previously described [13].

Immunoprecipitation assays were performed with 100 µg of SHEP cell extracts with 2 µg of rat or rabbit IgG2a, rat anti-LIMK2 [clone 1C6 [20]], rabbit anti-TPPP1 [42] and 50 µl of Protein A/G sepharose beads as previously described [44].

### Metabolic labeling of proteins

SHEP cells transiently transfected with GST-LIMK2a, GST-LIMK2b or the GST (pEBG) for 24 hours were treated for 10 hours with microtubule-targeted drugs. ^35^S-protein labeling was performed in the presence of the microtubule-targeted drugs as previously described [40].

### Immunoflourescence microscopy

Immunofluorescence was performed as previously described [45]. Cells were incubated with anti-LIMK2 [10 µg/ml, clone 1G128 [20]], anti-HA (1:400, Roche, 11867423001) or with anti-acetylated-tubulin (1:200, Sigma, T6793) followed by incubation with anti-mouse IgG Alexa 488 (1:400, Invitrogen) or anti-rat IgG Alexa 594 (1:400, Invitrogen) and Hoechst (1:10,000, Invitrogen). To visualize α-tubulin, cells were incubated with FITC-conjugated α-tubulin (1:200, Sigma, F2168). Images were acquired using either an Olympus Fluoview FV1000 confocal microscope (Olympus, GmbH) and FV10-ASW software (version 1.7.2.2; Olympus) or a fluorescent microscope (Olympus IX81 Live Cell Imager coupled to RETIGA EXi 32-0062B-173 cooled Mono 12 bit camera).

### Flow cytometry

For cell cycle analysis, cells were fixed with 70% ethanol overnight at 4°C, washed with PBS cells and incubated with propidium iodide staining buffer [10 mM Tris pH 7.5, 5 mM MgCl_2_, 20 µg/ml RNAse A and 5 µg/ml propidium iodide (Invitrogen)] for 30 minutes at 37°C. Flow cytometric analysis was carried out using a FACSCalibur flow cytometer (BD Biosciences) and CellQuest software. Cell cycle distribution was analyzed using FlowJo (v 8.8.6) software. On the displays shown, cells were gated to exclude debris and dead cells. For cell death quantification, cells were stained as above but without RNAse A.

To analyze apoptosis, cells were resuspended in AnnexinV binding buffer (10 mM HEPES pH 7.4, 140 mM NaCl and 2.5 mM CaCl_2_) and incubated with 1 µl of anti-AnnexinV Alexa 488 (Invitrogen) for 15 minutes at room temperature before addition of 1 µg/ml propidium iodide (Invitrogen). Flow cytometric analysis was performed using a FACSCalibur instrument (BD Biosciences).

### MTT assay

Cells were plated onto 96-well plates in triplicate and at each time point cells were incubated with MTT solution (CellTiter 96 AQueous Non-Radioactive Cell Proliferation Assay, Promega) at 37°C in 5% CO_2_ for 4 hours. Converted dye was measured using a spectophotometric plate reader (Titertek Multiskan Plus, Lab Systems).

### Quantitative PCR (qRT-PCR) Analysis

Total RNA was prepared from SHEP cells using the RNAeasy Mini Kit (Qiagen) followed by incubation with DNAse I (Promega). RNA concentration and purity was determined using a spectrophotometer (Nanodrop ND-1000 spectrophotometer, Thermo Scientific). 200 ng of total RNA were reversed-transcribed using Superscript II Reverse Transcriptase (Invitrogen) according to manufacturer’s instructions. qPCR was carried out in triplicate using SYBR green (Invitrogen) in a Mx3000P qPCR Machine from Strategene. Transcripts were normalized to the housekeeping gene L32. The oligonucleotide primers (Sigma) used were: LIMK2a forward 5’-GGGTGAAGATGTCTGGAG-3’; reverse 5’-TCGTTGACAGTCCTGTACC-3’. LIMK2b forward 5’-ATGGGGAGTTACTTGTCAGTC-3’; reverse 5’-CGAAACAGGTCTCTGGAG. L32 forward 5’-CAGGGTTCGTAGAAGATTCAAGGG-3’; reverse 5’-CTTGGAGGAAACATTGTGAGCGATC-3’.
